# Supplementary material for: The Role of Pretreatment Serum Interleukin 6 in Predicting Short-Term Mortality in Patients with Advanced Pancreatic Cancer
Source: Biomedicines. 2024 Apr 18;12(4):903. doi: 10.3390/biomedicines12040903 (PMC11048054; doi:10.3390/biomedicines12040903)
Supplement: Supplementary file 1 [file biomedicines-12-00903-s001.zip › biomedicines-2962167-supplementary.pdf]

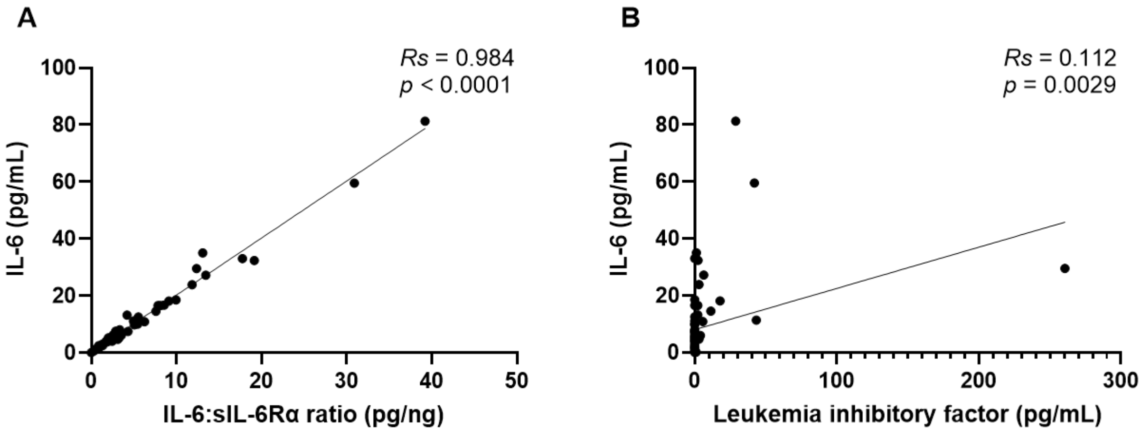

Figure. S1 Relationship between serum IL-6 and related biomarkers. (A) Correlation of serum IL-6 concentrations with the ratio of IL-6 to soluble IL-6 receptor. (B) Association between IL-6 concentrations and serum levels of leukemia inhibitory factor.

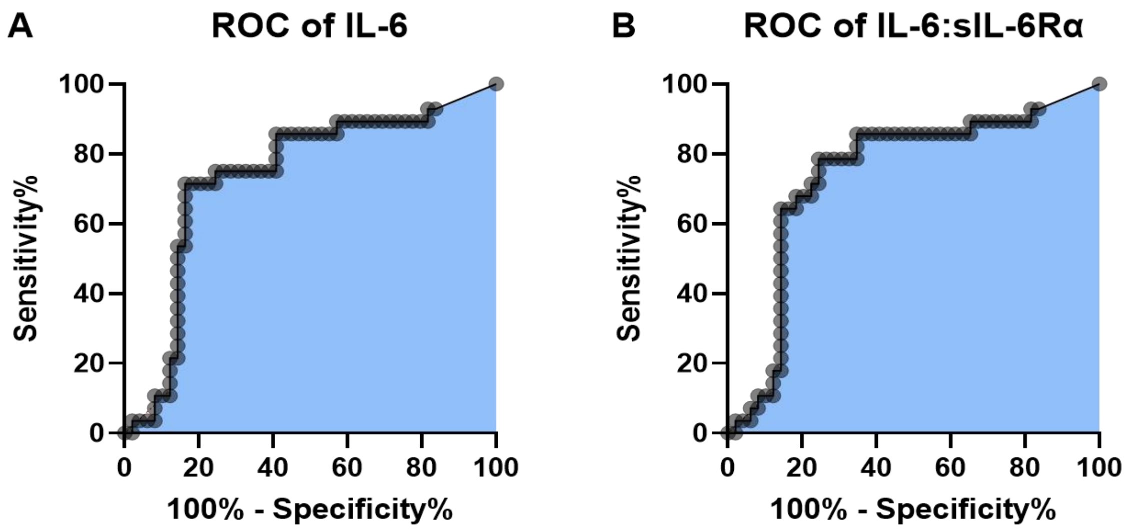

Figure. S2 Receiver operating characteristic (ROC) curve analysis to define an optimal prognostic cutoff value for predicting 6-month survival of patients with advanced pancreatic cancer. (A) ROC curve for serum interleukin-6 levels, (B) ROC curve for ratio of interleukin-6 to soluble interleukin-6 receptor.

Table S1. Efficacy of first-line systemic chemotherapy in patients with advanced pancreatic cancer categorized by serum IL-6 and soluble IL-6Rα ratio

Variables	Total (n=65)	IL-6:sIL-6Rα high (n=25)	IL-6:sIL-6Rα low (n=40)	*p* value
**First-line chemotherapy, n (%)**				
Gemcitabine-based	41 (63.1)	20 (80.0)	21 (52.5)	0.025
Gemcitabine single	5 (7.7)	5 (20.0)	0	
Gemcitabine / Nab-paclitaxel	36 (55.4)	15 (60.0)	21 (52.5)	
FOLFIRINOX	24 (36.9)	5 (20.0)	19 (47.5)	
**Best response, n (%)**				
Partial response	11 (16.9)	3 (12.0)	8 (20.0)	
Stable disease	32 (49.2)	8 (32.0)	24 (60.0)	
Progressive disease	22 (33.9)	14 (56.0)	8 (20.0)	
**Objective response rate, n (%)**	11 (16.9)	3 (12.0)	8 (20.0)	0.509
**Disease control rate, n (%)**	43 (66.2)	11 (44.0)	32 (80.0)	**0.003**
**Median PFS, months [95% CI]**	5.9 [4.8–7.0]	3.1 [0.5–5.7]	9.1 [6.8–11.4]	<0.001
**6-months PFS, % [95% CI]**		21.7 [8.0–39.7]	63.9 [46.7–81.1]	

IL-6 interleukin-6, IL-6Rα interleukin-6 receptor α subunit, *FOLFIRINOX* fluorouracil, leucovorin, irinotecan, and oxaliplatin, *PFS* progression-free survival.
